# Evaluating toric intraocular lens outcomes: A comparison between asymmetric and symmetric bowtie astigmatism

**DOI:** 10.1371/journal.pone.0362262

**Published:** 2026-11-13

**Authors:** Natpachara Emmaneerat, Lita Uthaithammarat, Thanapong Somkijrungroj, Vannarut Satitpitakul

**Affiliations:** 1 Faculty of Medicine, Department of Ophthalmology, Chulalongkorn University, Bangkok, Thailand; 2 Center of Excellence in Retina, Department of Ophthalmology, Faculty of Medicine Chulalongkorn University and King Chulalongkorn Memorial Hospital, Thai Red Cross Society, Bangkok, Thailand; 3 Center of Excellence for Cornea and Stem Cell Transplantation, Department of Ophthalmology, Faculty of Medicine, Chulalongkorn University, Bangkok, Thailand; 4 Excellence Center for Cornea and Stem Cell Transplantation, Department of Ophthalmology, King Chulalongkorn Memorial Hospital, Thai Red Cross Society, Bangkok, Thailand; Akita University: Akita Daigaku, JAPAN

## Abstract

**Objectives:**

To compare outcomes of toric intraocular lens (IOL) implantation in patients with asymmetric versus symmetric bowtie astigmatism.

**Methods:**

This retrospective chart review included 76 eyes from 60 patients: 24 eyes (20 patients) with asymmetric bowtie astigmatism (Group 1) and 52 eyes (43 patients) with symmetric bowtie astigmatism (Group 2). All patients underwent uneventful phacoemulsification with toric IOL implantation. Uncorrected distance visual acuity (UCVA), residual astigmatism, and spectacle independence at three months postoperatively were evaluated.

**Results:**

The median UCVA was 0.10 logMAR in Group 1 and 0.00 in Group 2 (P = 0.045). UCVA of 20/20 or better was achieved in 29% of Group 1 and 52% of Group 2. The median residual astigmatism was significantly higher in Group 1 (−1.39 D, IQR 1.23) than in Group 2 (−0.70 D, IQR 0.35; P < 0.01). Spectacle-free rates were 88.89% in Group 1 and 81.82% in Group 2 (P = 0.709).

**Conclusion:**

At three months postoperatively, toric IOL implantation provided comparable UCVA and spectacle independence in both groups, though residual astigmatism was significantly higher in the asymmetric group.

## Introduction

Astigmatism and cataracts significantly reduce an individual's quality of life, by affecting visual acuity and daily activities [1]. Michlitsch et al. reported that the prevalence of corneal astigmatism greater than 1.0 diopter (D) among cataract surgery in Europe ranges from 30% to 39% [2]. In China, nearly half (47.27%) of preoperative eyes in the northern regions exhibit corneal astigmatism of 1.0 D or more, while over 40% of cataract surgery candidates in India have similar degrees of astigmatism, suggesting a large patient population that may benefit from toric intraocular lens (IOLs) [3, 4]

In contemporary cataract surgery, achieving spectacle independence has emerged as a critical goal for both patients and surgeons. Failure to correct corneal astigmatism during this procedure can result in 20% to 30% of patients remaining reliant on spectacles postoperatively [5]. To address this challenge, toric IOLs have gained recognition for their ability to deliver optimal visual outcomes and reduce spectacle dependence. Clinical evidence supports the safety and efficacy of toric IOLs implantation for cataract patients with low-to-high symmetrical bowtie corneal astigmatism, establishing them as a safe and effective treatment option for such cases [6,7].

Among various corneal topographic patterns, Kanpolat et al. found that asymmetric bowtie astigmatism (33%) is the most common pattern, followed by symmetric bowtie (29%), round (14%), irregular (12%), and oval (11%) patterns [8]. Round and oval patterns represent little or mild corneal astigmatism, whereas bowtie patterns indicate clinically significant regular astigmatism that is typically suitable for toric intraocular lens (IOL) correction. In general, toric IOLs are ideally used in patients with symmetrical bowtie astigmatism, while using toric IOLs in patients with asymmetrical bowtie astigmatism might lead to uncertain visual outcomes. Despite the large number of patients, the efficacy of toric IOLs in correcting asymmetric bowtie astigmatism remains unclear.

In this study, we aimed to compare the visual outcomes, residual astigmatism, and spectacle-free rate for distance vision in patients with asymmetric and symmetric bowtie astigmatism following uneventful phacoemulsification and toric IOL implantation.

## Methods

This retrospective chart review was conducted in patients who underwent uneventful cataract surgery at King Chulalongkorn Memorial Hospital (KCMH). The study was approved by the Institutional Review Board of the Faculty of Medicine, Chulalongkorn University, Bangkok, Thailand (COA No. 1532/2023), in accordance with international ethical guidelines for human research protection, including the Declaration of Helsinki, the Belmont Report, CIOMS guidelines, and the International Conference on Harmonization Good Clinical Practice.

The Institutional Review Board (IRB) waived the requirement for written informed consent for the retrospective chart review, and all data were de-identified prior to analysis. For the telephone interview, patients were first informed about the study by their treating ophthalmologist and subsequently contacted by a study investigator, who explained the study using a standardized script. Verbal informed consent was obtained before the interview, and the patient’s consent was documented in the study records by the investigator. The use of verbal consent and its documentation procedure were reviewed and approved by the Institutional Review Board.

The inclusion criteria were as follows: patients aged 18 years or older with bowtie corneal astigmatism 0.75 D or more who underwent uneventful phacoemulsification with toric IOLs implantation at KCMH. Eligible patients had a complete ophthalmic examination, including automated refractometry and corneal topography using Pentacam HR (Oculus, Wetzlar, Germany). Patients with bowtie corneal astigmatism, defined by an I-S difference greater than 1.5 D or an S-I difference greater than 2.5 D within the central 5-mm zone, along with a skewed radial axis (SRAX) not more than 22 degrees, were assigned to the asymmetrical bowtie group [9,10]. The other patients with bowtie corneal astigmatism were placed in the symmetrical bowtie group. Exclusion criteria consisted of patients with corneal diseases, advanced glaucoma, retinal or macular diseases, a history of previous refractive surgery or corneal transplantation, postoperative complications such as toxic anterior segment syndrome or endophthalmitis, other ocular conditions that could affect visual function, and eyes with incomplete postoperative data at the three-month follow-up.

Data on uncorrected distance visual acuity (UCVA), corrected distance visual acuity (CDVA), and residual astigmatism, were collected preoperatively and at three months postoperatively. Spectacle use was assessed through a telephone interview, following an informed consent script. All data were collected during March to September 2024.

All surgeries were performed by two experienced surgeons (VS and TS) between December 2021 and February 2024. In brief, topical anesthesia was applied, and a 2.75 mm clear corneal incision was made. Phacoemulsification was performed, and a toric IOL was implanted in the capsular bag, aligned with the intended axis, which was marked using an intraoperative toric marker at the corneal limbus (Callisto or Verion^TM^ Image Guided System, Alcon Laboratories, Inc., Fort Worth, TX, USA). Toric intraocular lenses calculated by a standard industry calculator using front keratometry values. No corneal suture was placed. Postoperatively, a combination of tobramycin 0.3% (3 mg) and dexamethasone 0.1% (1 mg) eye drops were administered four times daily for 30 days.

Preoperative biometry, including anterior corneal keratometry (K1 and K2), was obtained using the IOLMaster 700 (Carl Zeiss Meditec, Jena, Germany). These anterior K values, rather than total or true net corneal power, were entered into the Barrett Toric Calculator, accessed via the Asia-Pacific Association of Cataract and Refractive Surgeons (APACRS) online platform (calc.apacrs.org), to determine IOL cylinder power and the predicted implantation axis. Pentacam HR tomography was used separately, as described above, to classify eyes into symmetric and asymmetric bowtie groups, and was not used as an input for IOL power calculation.

The efficacy index was calculated as the ratio of the mean postoperative uncorrected distance visual acuity (UCVA) to the mean preoperative corrected distance visual acuity (CDVA). The safety index was calculated as the ratio of the mean postoperative CDVA to the mean preoperative CDVA. These indices reflect the effectiveness and safety of the surgical procedure, respectively [11].

Data was stored in Microsoft Excel, and statistical analysis was performed using Stata version 18. (StataCorp, College Station, TX, USA). Descriptive statistics were calculated, with categorical data presented as percentages, continuous data with normal distribution shown as means with standard deviations, and non-normal distribution data presented as medians with interquartile ranges. The Wilcoxon rank-sum (Mann–Whitney U) test was used to compare non-normally distributed continuous variables between the two groups, and Fisher's exact test was used to compare proportions between groups. The pupil size cut-off points were identified using Youden’s index to predict post-operative UCVA equal or better than Snellen 20/25. The pupil thresholds and absolute value of post-operative astigmatism were used for further logistic regression analysis. A p-value of less than 0.05 was considered statistically significant.

## Results

A total of 76 eyes were included in the analysis. Of these, 24 eyes (31.6%) exhibited asymmetric bow-tie astigmatism, while 52 eyes (68.4%) had symmetric bow-tie astigmatism. Demographic data is presented in Table 1. Within the asymmetric group, 6 eyes (25%) displayed a S-I pattern, and 18 eyes (75%) showed an I-S pattern.

**Table 1:** Demographic Data.

	Asymmetrical bowtie astigmatism group (N = 24 eyes)	Symmetrical bowtie astigmatism group (N = 52 eyes)
Age, years (means ± SD)	67.75 ± 11.25	69.58 ± 8.24
Sex, No. of female (%)	18 (75.0%)	30 (57.7%)
Laterality, No. of right eye (%)	16 (64.0%)	25 (47.17%)
Pre-operative astigmatism, diopters [median (IQR)]	−2.13 (2.50)	−1.63 (1.13)
Pre-operative axis of steepest cornea at 3-mm zone, Degrees [median (IQR)]	85^∘^ (55.5)	94^∘^ (139.5)
Skew angle at 3-mm zone, Degrees [median (IQR)]		
	1.5^∘^ (8)	5^∘^ (11)
Pre-operative axis of steepest cornea at 5-mm zone, Degrees [median (IQR)]		
	91.5^∘^ (40)	113^∘^ (114)
Skew angle at 5-mm zone, Degrees [median (IQR)]		
	3.5^∘^ (10)	8^∘^ (14.5)
Pre-operative uncorrected distance visual acuity, logMAR [median (IQR)]		
	0.40 (0.69)	0.35 (0.49)
Pre-operative corrected distance visual acuity, logMAR [median (IQR)]		
	0.30 (0.35)	0.20 (0.30)
IOL spherical power, diopters [median (IQR)]		
	19.00 (3.75)	19.00 (3.50)
IOL cylinder power, diopters		
	2.13 (2.00)	2.00 (1.25)
Pupil diameter, mm (mean ± SD)	2.49 ± 0.60	2.81 ± 0.80
IOL types Monofocal toric AT TORBI (Zeiss) DIU (J&J)		
	
	15 (62.5%)	21 (40.38%)
	0 (0.00%)	1 (1.92%)
EDOF toric DFT (Alcon)		
	2 (8.33%)	7 (13.46%)
Multifocal toric		
ATLISA toric (Zeiss)	1 (4.17%)	9 (17.31%)
TFNT (Alcon)	5 (20.83%)	12 (23.08%)
CNWTT (Alcon)	0 (0.00%)	2 (3.85%)
LU313MFT (Teleon)	1 (4.17%)	0 (0.00%)

All postoperative outcomes reported below were assessed three months after phacoemulsification and toric IOL implantation. The mean postoperative spherical equivalents showed no significant difference, with values of −0.51 D (SD 0.81) in asymmetric group and −0.24 D (SD 0.65) in symmetric group (P = 0.13). The post-operative UCVA, CDVA, and residual astigmatism is shown in Table 2. Median UCVA and residual astigmatism were significantly better in symmetric group (P = 0.045 and <0.01, respectively).

**Table 2:** Comparison of UCVA, CDVA, and residual astigmatism at 3 months postoperatively between asymmetric bowtie and symmetric bowtie groups.

	Asymmetric Bowtie Astigmatism Group	Symmetric Bowtie Astigmatism Group	P-value
UCVA, logMAR [median (IQR)]	0.10 (0.20)	0.00 (0.10)	0.045*
CDVA, logMAR [median (IQR)]	0.01 (0.10)	0.00 (0.10)	0.63
Residual Astigmatism, diopters (median ± IQR)	−1.39 ± 1.23	−0.70 ± 0.35	< 0.01*

*Wilcoxon rank-sum (Mann–Whitney U) test

The cumulative post-operative Snellen visual acuity was shown in Fig 1. In the asymmetric group, 7 of 24 eyes (29.17%) and in the symmetric group, 27 of 52 eyes (51.92%) achieved UDVA of 20/20 or better. CDVA of 20/20 or better was achieved in 13 of 24 eyes (54.17%) in the asymmetric group and in 29 of 52 eyes (55.77%) in the symmetric group. Difference between groups was not statistically significant for UDVA (P = 0.06) or CDVA (P = 0.90).

**Fig 1:**
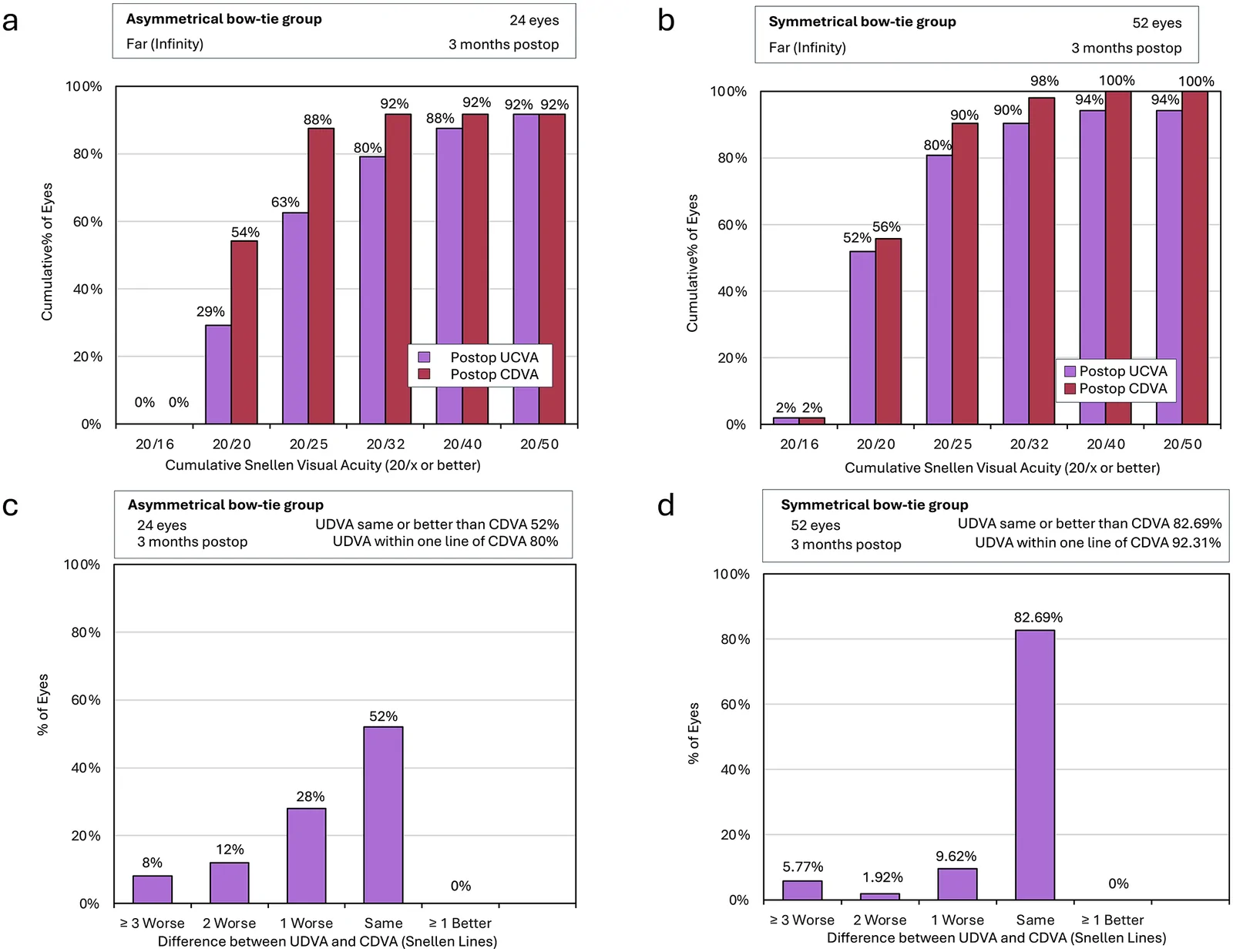
Efficacy outcomes of asymmetrical and symmetrical bowtie groups at 3 months post-cataract surgery. Cumulative percentage of eyes achieving monocular UDVA and CDVA in (a) asymmetrical bowtie group and (b) symmetrical bowtie group. Difference between UDVA and CDVA in Snellen lines in (c) asymmetrical bowtie group and (d) symmetrical bowtie group.

### Efficacy outcomes

Postoperatively, the proportion of eyes with UDVA equal to or better than CDVA was significantly lower in the asymmetric group (52.0%) compared to the symmetric group (81.4%) (P = 0.01). The proportion with UDVA within one line of CDVA did not differ significantly (P = 0.12) (Fig 1). Median efficacy index was 1.58 (IQR 1.26) in the asymmetric group and 1.25 (IQR 0.80) in the symmetric group, with no significant difference (P = 0.44).

### Predictability outcomes

As shown in Fig 2, postoperative spherical equivalent refraction accuracy within ± 0.50 D was achieved in 62.50% of eyes in the asymmetric group and 63.46% in the symmetric group (P = 0.94). Within ±1.00 D, accuracy was 86.84% and 88.46% respectively (P = 0.72). The mean residual astigmatism was significantly higher in the asymmetric group (−1.39 ± 1.23 D) compared with the symmetric group (−0.70 ± 0.35 D, P < 0.01). As shown in Fig 2, residual astigmatism ≤ −0.50 D was achieved in 16.7% of asymmetric eyes versus 42.3% of symmetric eye (P = 0.04). Residual astigmatism ≤ −1.0 D was observed in 66.7% of asymmetric eyes and 84.6% of the symmetric eyes (P = 0.07).

**Fig 2:**
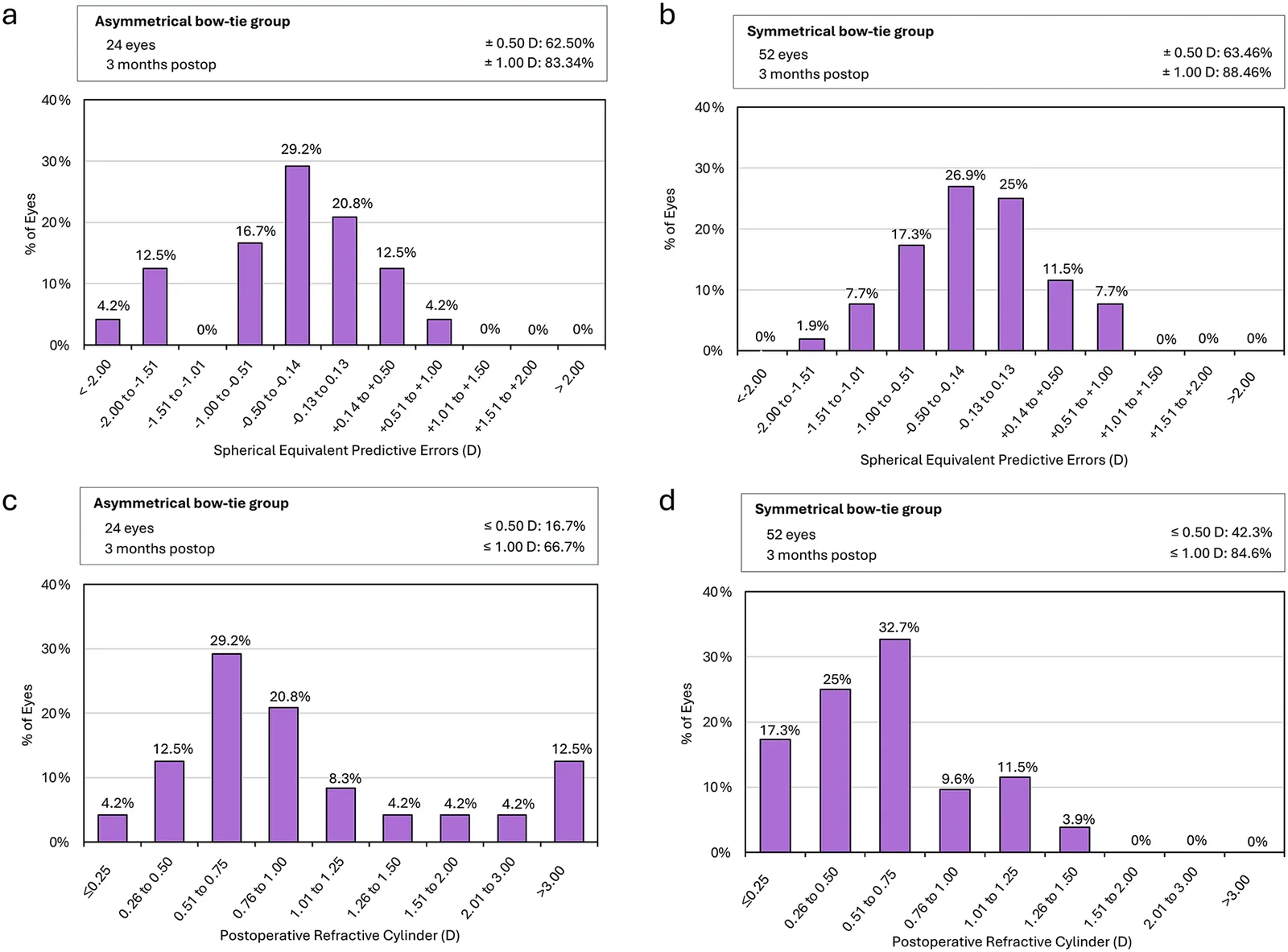
Predictability outcomes of asymmetrical and symmetrical bowtie groups at 3 months post-cataract surgery. Spherical equivalent predictive errors in (a) asymmetrical bowtie group and (b) symmetrical bowtie group. Post-operative refractive cylinder in (c) asymmetrical bowtie group and (d) symmetrical bowtie group.

### Safety outcomes

The median safety index was 1.6 (IQR 1.0) in the asymmetric group and 1.28 (IQR 1.0) in the symmetric group (P = 0.24). Loss of ≥ 2 lines of CDVA occurred in 0% of asymmetric eyes and 1.89% of symmetric eyes, with no significant difference (P = 1.00) (Fig 3).

**Fig 3:**
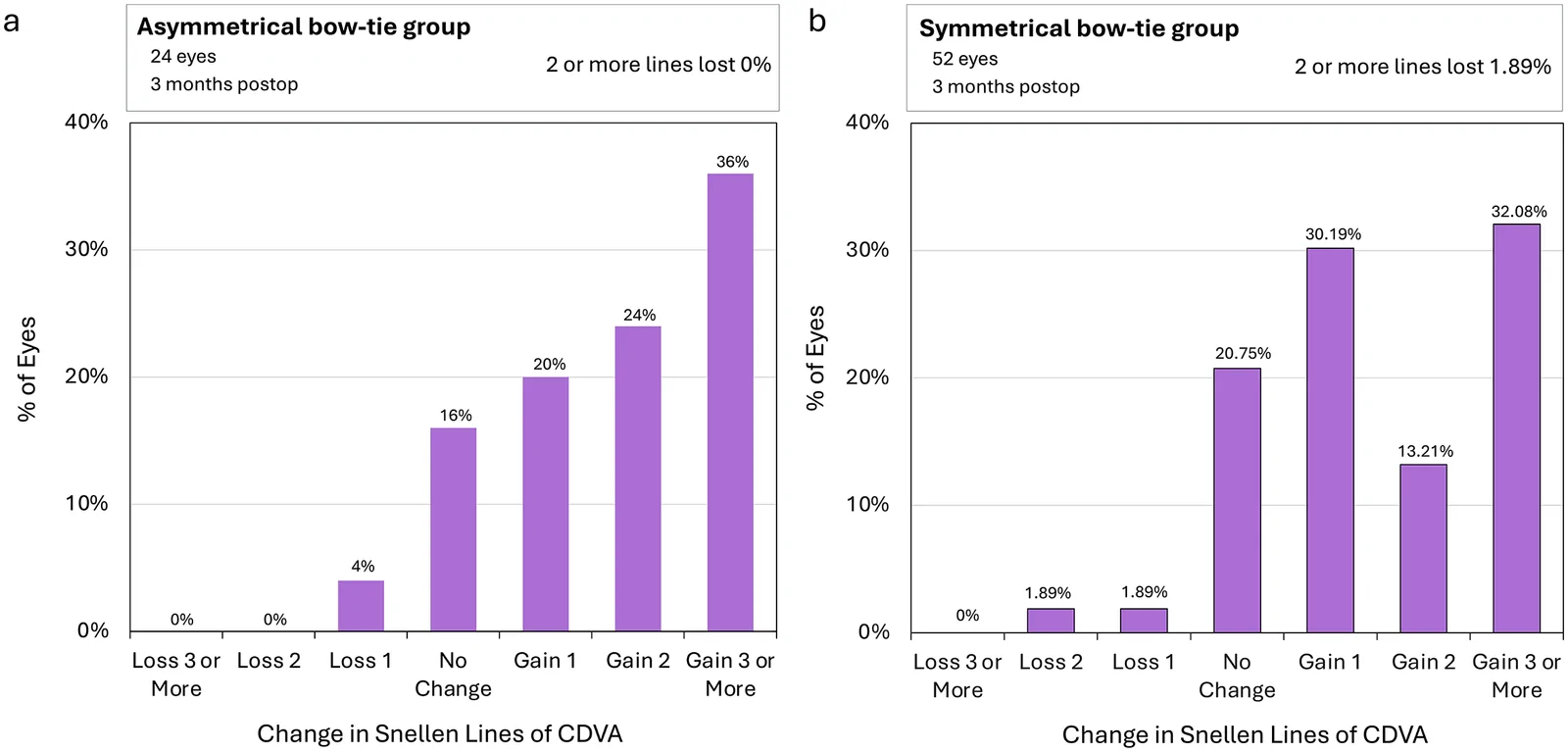
Safety outcomes of asymmetrical and symmetrical bowtie groups at 3 months post-cataract surgery. Changes in Snellen lines of CDVA in (a) asymmetric and (b) symmetric groups.

### Spectacle free rates

Spectacle independence was similar between groups, achieved in 88.89% of asymmetric eyes (16/18; 6 missing data) and 81.82% of symmetric eyes (36/44; 8 missing data), with no significant difference (P = 0.71).

### Pupil effect on visual acuity

Youden’s index identified the optimal cut-off values for pupil size of ≥2.3 mm in the asymmetric group and ≥2.5 mm in the symmetric group. In the asymmetric group, pupil size ≥2.3 mm was not significantly associated with postoperative UDVA better than 20/25 in either binary logistic regression (OR = 1.38, 95% CI: 0.23–8.30, P = 0.73) or multivariate analysis adjusting for postoperative UDVA, pupil size (≥2.3 mm), and postoperative cylinder (OR = 2.34, 95% CI: 0.33–16.88, P = 0.40). Similarly, absolute postoperative cylinder was not a significant predictor (OR = 1.85, 95% CI: 0.82–4.15, P = 0.14).

In the symmetric group, binary logistic regression showed that pupil size ≥2.5 mm was significantly associated with postoperative UDVA better than 20/25 (OR = 5.83, 95% CI: 1.29–26.38, P = 0.02). Multivariate analysis confirmed this association (OR = 7.23, 95% CI: 1.20–43.43, P = 0.03) and additionally identified absolute postoperative cylinder as a significant predictor (OR = 0.03, 95% CI: 0.001–0.42, P = 0.01).

## Discussion

Previous studies have reported favorable outcomes of toric IOL implantation in eyes with asymmetric corneal astigmatism, but direct comparisons with symmetric astigmatism remain limited [12–15]. In this study, we compared efficacy, predictability, and safety between eyes with symmetric and asymmetric bowtie astigmatism after phacoemulsification and toric IOL implantation. Our findings demonstrate that while both groups achieved substantial improvements in visual acuity and spectacle independence, residual astigmatism was significantly higher in the asymmetric group.

At three months, eyes with symmetric astigmatism achieved significantly better median UCVA and lower residual astigmatism compared with asymmetric eyes. These findings suggest that toric IOLs correct symmetric astigmatism more predictably. Notably, the mean residual astigmatism in our asymmetric cohort (1.39 D) was higher than values previously reported (0.65–0.87 D) [12,14–16]. Nevertheless, postoperative BCVA, cumulative Snellen visual acuity, efficacy index, and spectacle independence were comparable between groups, indicating that residual cylinder might not translate into clinically meaningful differences in visual function. Patients with asymmetric astigmatism still achieved excellent unaided vision overall.

Our results are consistent with prior reports. Li et al. evaluated asymmetric bowtie patterns at the 5-mm zone and reported favorable outcomes [15], while Gao et al. included both asymmetric and angled bowtie patterns at the 3-mm zone [14]. Chen et al. found that eyes with angled bowtie astigmatism (SRAX 30–45°) achieved higher refractive accuracy than those with asymmetric bowtie (SRAX <30°), highlighting the influence of astigmatism classification [16]. Importantly, our classification was based on the 5-mm central zone, which may capture more peripheral irregularities relevant to IOL alignment. Indeed, some patients categorized as asymmetric in the 5-mm zone showed angled features at the 3-mm zone, underscoring variability across classification systems.

Predictability, assessed by postoperative spherical equivalent within ±0.50 D and ±1.00 D, did not differ significantly between groups. Safety outcomes were similarly favorable in both groups, with minimal risk of CDVA loss. No eyes in the asymmetric group lost ≥2 lines of vision, supporting the overall safety of toric IOL implantation in these cases. One eye in the symmetric group lost two lines of CDVA (20/20–20/30) postoperatively. This eye's calculator-predicted residual cylinder was −0.08 D at axis 5°, but the postoperative manifest refraction was −0.25/ − 1.00 × 138°—a substantial deviation from the predicted target in both cylinder magnitude and axis. This apparent discrepancy is consistent with toric IOL misalignment or postoperative rotation, though it may also reflect a shift in effective lens position or a change in corneal astigmatism between the preoperative and postoperative measurements. As postoperative toric axis position was not independently verified in this series, we are unable to confirm actual IOL axis malposition, and this finding should be interpreted as a refractive outcome rather than direct evidence of lens rotation.

An additional aspect of our study was the evaluation of pupil size. In the symmetric group, larger pupil size (≥2.5 mm) and lower residual astigmatism were associated with better postoperative UDVA, consistent with prior reports that optimal image quality is achieved with pupils of 2–3 mm [17–20]. By contrast, in the asymmetric group, pupil size and residual astigmatism were not significantly associated with visual outcomes. This discrepancy may reflect the greater contribution of higher-order aberrations, such as coma, in asymmetric astigmatism. Indeed, previous studies have shown that total HOAs are significantly higher in asymmetric bowtie eyes [21], which may explain the weaker association between pupil metrics and vision in this subgroup.

This study has some limitations. First, this was a retrospective series and did not include systematic postoperative verification of actual IOL axis position (e.g., slit-lamp toric marker alignment or photographic/Purkinje-image-based analysis against the intended axis). We therefore used postoperative manifest refraction-derived residual astigmatism as a clinical surrogate for net toric outcome; while this is a common approach when direct axis-marker verification is not performed, it does not allow us to distinguish how much of the residual astigmatism—particularly the larger residual astigmatism observed in the asymmetric group—reflects true intraoperative axis misalignment or postoperative lens rotation versus other contributing factors, such as posterior corneal astigmatism, effective lens position, or biometric variability between groups. Second, the three-month follow-up, although useful for evaluating short-term outcomes, may not fully capture the long-term stability of visual acuity and refractive results following toric IOL implantation, including the possibility of late lens rotation. Third, the study did not assess higher-order aberrations or provide detailed corneal classification methods beyond the central 5-mm zone, both of which could influence the interpretation of surgical outcomes. In particular, higher-order aberrations such as coma and trefoil, which are more prominent in asymmetric bowtie corneas, [21] may contribute to the refractive astigmatism measured postoperatively and cannot be corrected by a toric IOL; future studies incorporating preoperative and postoperative wavefront aberrometry should therefore quantify the contribution of higher-order aberrations to residual astigmatism and visual outcomes in these eyes. Prospective studies incorporating postoperative axis photography or dedicated toric alignment analysis, longer follow-up, and aberrometric assessment are warranted to clarify these relationships further.

In summary, toric IOL implantation provides excellent safety and visual outcomes in eyes with both symmetric and asymmetric bowtie astigmatism. While symmetric eyes achieved lower residual astigmatism and slightly better uncorrected vision, asymmetric eyes also attained high rates of spectacle independence and comparable functional outcomes.
